# Categorical processing of fast temporal sequences in the guinea pig auditory brainstem

**DOI:** 10.1038/s42003-019-0472-9

**Published:** 2019-07-19

**Authors:** Alice Burghard, Mathias Benjamin Voigt, Andrej Kral, Peter Hubka

**Affiliations:** 1 Institute of Audioneurotechnology & Department of Experimental Otology, ENT Clinics, Hannover Medical School, Hannover, D-30625 Germany; 2 Department of Neuroscience, University of Connecticut Health Center, Farmington, CT 06030 USA

**Keywords:** Auditory system, Sensory processing, Perception

## Abstract

Discrimination of temporal sequences is crucial for auditory object recognition, phoneme categorization and speech understanding. The present study shows that auditory brainstem responses (ABR) to pairs of noise bursts separated by a short gap can be classified into two distinct groups based on the ratio of gap duration to initial noise burst duration in guinea pigs. If this ratio was smaller than 0.5, the ABR to the trailing noise burst was strongly suppressed. On the other hand, if the initial noise burst duration was short compared to the gap duration (a ratio greater than 0.5), a release from suppression and/or enhancement of the trailing ABR was observed. Consequently, initial noise bursts of shorter duration caused a faster transition between response classes than initial noise bursts of longer duration. We propose that the described findings represent a neural correlate of subcortical categorical preprocessing of temporal sequences in the auditory system.

## Abstract (web summary)

Alice Burghard et al. record auditory brainstem responses from guinea pigs during the presentation of paired noise bursts separated by a short gap. The responses obtained can be categorized into two groups based on the leading noise burst and the following gap duration, suggesting that categorical processing of temporal sequences can occur already in the brainstem.

## Introduction

Categorical perception is one of the basic principles that drive cognition and behavior^1,2^. It enables the differentiation of distinct classes in sensory perception when incoming sensory information changes gradually along a continuum. Categorization corresponds to a nonlinear transformation of a linearly changing variable into a neuronal representation enabling rapid transition between categories^1^. Categorical grouping of sensory information represents an efficient adaptive tool for coping with a constantly changing and complex world. It allows generalization of sensory features, construction of internal sensory objects, and, thus, enhanced detection and encoding of familiar as well as novel sensory objects^3–6^.

Categorical perception was first introduced in studies on speech perception and auditory processing in humans^7,8^. Specifically, it defines phoneme categories with sharp transitions between the perception of consonants-vowel pairs (e.g,. pa-ba, ta-da, and ka-ga) when the timing between the stop consonant and vowel, called voice-onset time, continuously changes^7,9^. This demonstrates that temporal coding plays an important role in the categorical coding of these phonemes.

Vocal communication in animals has reliably been shown to be based on categorical perception^10^, including voice-onset time dependent classification (chinchilla^11^; monkey^12^). It is generally assumed that the neuronal processing underlying categorical perception is accomplished in the cerebral cortex^13,14^. Indeed, activity in several cortical areas was identified to mirror categorization in the perception of sounds^15–17^. Previous studies have, however, provided evidence that the encoding of temporal patterns of acoustical signals is already performed at the level of the auditory brainstem in humans^18^. Temporal response precision in the auditory brainstem has also been shown to be related to speech perception^19,20^ and reading skills^21^. This link between temporal response precision and speech perception indicates that the neuronal coding of temporal structure is involved in the auditory categorical perception and can start as early as in the auditory brainstem. To verify this hypothesis, an analysis of neuronal responses from the auditory brainstem to a systematically changing temporal structure is necessary.

The present study approaches this problem by analyzing auditory brainstem responses (ABR) to changes in the temporal structure of auditory stimuli, which contain no relevant spectral information. We show that the temporal pattern of an auditory stimulus is nonlinearly transformed into auditory brainstem activation. This transformation could provide an important subcortical contribution to the final neuronal response categorization in the cerebral cortex.

## Results

**Fig. 1:**
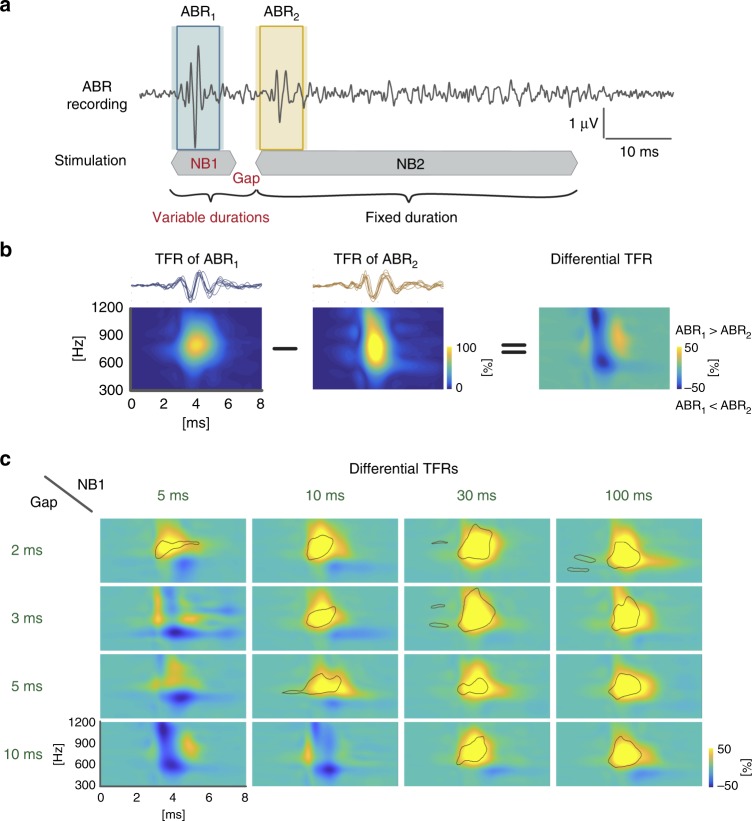
Combination of leading noise burst and gap duration determines time-frequency representations of the onset ABR to the trailing noise burst. **a** Schematic representation of stimulus sequence together with the matched recorded ABR signals. Leading noise burst (NB1) and gap duration were systematically varied (NB1 durations: 5, 10, 30, and 100 ms; gap durations: 2, 3, 5, and 10 ms). The duration of the trailing noise burst (NB2) was fixed at 50 ms. The shaded boxes represent the first 8 ms post-stimulus time used for TFR analysis. The frame represents a time window of 6 ms duration (latencies of 1–7 ms) used for quantitative time-domain analysis (Supplementary Fig. 1). For details, see Methods. **b** Example of time-frequency representations (TFR) of the ABRs (NB1 duration = 5 ms; gap duration = 10 ms) and the differential TFR between the leading and trailing onset ABRs (for details, see Methods). **c** Grand means of differential TFRs for all recorded combinations of NB1 and gap duration. Yellow and blue colors represent higher power in ABR_1_ and ABR_2_, respectively. Green color represents no change in power between ABRs. Black contour lines indicate statistically significant difference between TFR to leading and trailing noise bursts

We studied the neuronal representation underlying early phases of auditory processing in the brainstem of guinea pigs based on the temporal pattern of the acoustic stimulation. For this purpose, the auditory brainstem responses (ABRs) to initial noise bursts (NB1, duration of 5–100 ms) and trailing noise bursts (NB2, duration 50 ms) separated by short gaps (2–10 ms) were recorded (Fig. 1a). Random noise stimuli were used to avoid any informative spectral cues. This allowed us to investigate the impact of the temporal pattern of the stimulus (NB1-GAP-NB2 sequence) on ABR characteristics, per se.

We did not find any significant difference in the onset ABRs evoked by the initial NB1 (ABR_1_) for all conditions tested. Onset ABRs evoked by the trailing NB2 (ABR_2_), however, varied depending on the NB1 duration and gap duration (see the raw data and group time-domain representation in Supplementary Figs. 1 and 2, respectively).

### Time-frequency representation of ABRs

The ABRs to stimulus onsets were analyzed using their time-frequency representations (TFR). The TFR of the ABR signal was used for its explicit access to the information about the power of any given frequency range. Consequently, the TFR provides information about the time course of the single frequencies. Furthermore, power changes in several frequency ranges usually coexist and can be better identified using this analysis. The TFR analysis is therefore an excellent tool to study such co-activations.

The ABR signals were dominated by frequencies between 650–900 Hz (corresponding to wave period durations of 1.5–1.1 ms; Fig. 1b). The lower and the higher frequency bands (LFB: 400–650 Hz, and HFB: 900–1150 Hz, respectively) represent slower waves (wave period of 2.5–1.5 ms), on which the main waves are superimposed, and faster waves (wave period of 1.1–0.9 ms), which mirror a sharpening of the waves of the main signal, respectively. The LFB and HFB thus represent important information about a main ABR signal modulation making them good candidates for further analysis.

### Identification of two groups based on stimulus sequence

In order to identify specific effects of the initial response on the trailing response in a fast sequence, the differences between TFRs of ABR_2_ and ABR_1_ were computed (Fig. 1b). Positive power of differential TFRs (hot colors in the plots) indicated a larger power of corresponding frequencies in responses to NB1. Negative differential TFR power (cold colors) indicated larger powers in responses to NB2.

The differential TFRs seem to fall into two qualitatively different groups (Fig. 1c):

1. Balanced/enhanced group—This group was characterized by ABR_2_ ≥ ABR_1_; it comprised sequences of brief initial noise bursts (durations of 5 and 10 ms) followed by a gap of 3–10 ms and 10 ms, respectively;
2. Suppressed group—In this group, ABR_2_ < ABR_1_; it comprised all other stimulation sequences.

The balanced/enhanced group exhibited no change in the dominant frequency range (650–900 Hz), but a slight increase of power in the lower and the higher frequency bands in ABR_2_ compared to ABR_1_ (blue areas in the TFR color plots in Fig. 1c). The suppressed group was characterized by a suppression of ABR_2_ power in the dominant frequency range compared to ABR_1_ (yellow central areas in the TFR color plots in Fig. 1c; compare to Supplementary Fig. 1 for time-domain representations).

**Fig. 2:**
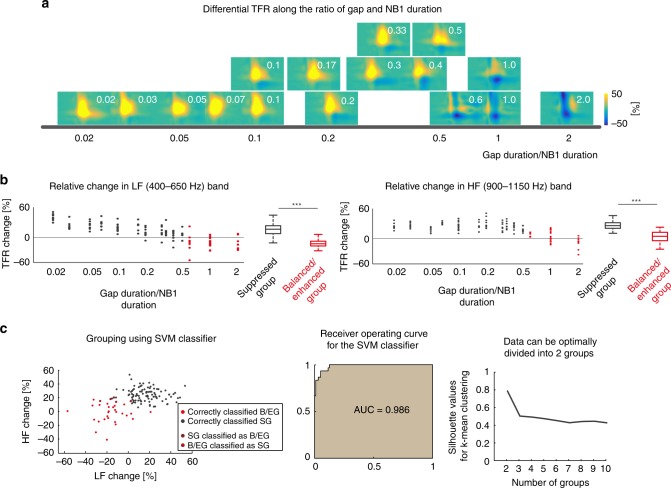
Classification of the ABR signals based on the ratio between gap and leading noise burst duration. **a** Temporal sequence of leading noise burst (NB1) and gap can be characterized by a ratio of gap duration and NB1 duration (see Table 1). Differential TFRs can be organized along this variable (depicted in upper right corner of each TFR) indicating an abrupt transition between the observed groups at the ratio of gap duration and NB1 duration between 0.5 and 0.6. The vertical arrangement of the single TFRs is to avoid overlap in conditions with similar gap/NB1 duration ratios and contains no additional information. **b** Relative power of TFR differences between ABR_1_ and ABR_2_ in low and high frequency bands (LFB—400–650 Hz and HFB—900–1150 Hz, respectively) for latencies of 3–5 ms showed a significant separation between the two identified groups along the ratio of gap duration and NB1 duration. In the left panels, the data are shown for all gap duration and NB1 duration ratios (*n* = 8); grey symbols represent the Suppressed group, red symbols the Balanced/Enhanced group. The data with the ratio of gap duration and NB1 duration of 1 were identical for 2 conditions (gap duration—5 ms and NB1 duration—5 ms; gap duration—10 ms; and NB1 duration—10 ms) and were pooled together (*n* = 16 and 14, respectively). In the right panels, boxes indicate twenty-fifth and seventy-fifth quartile around the median, the whiskers represent the data range (*p* = 1.1 × 10^−13^ for LF band, *p* = 4.5 × 10^−14^ for LF band; suppressed group *n* = 96, balanced/enhanced *n* = 30). **c** Left panel: Grouping of data low and high frequency change in TFR from the time window (latency of 3–5 ms) according to the Support Vector Machine (SVM) analysis; gray symbols represent the Suppressed group (SG), red symbols the Balanced/Enhanced group (B/EG); single-colored dots represent correctly classified data points, double-colored dots represent data points, which were misclassified. Middle panel: Receiver operating characteristic curve shows high and robust classification reliability based on the SVM model after 10-fold cross-validation (AUC Area under curve); Right panel: Result of the Silhouette analysis confirms optimal data classification into two groups

Since transitions between the identified groups depended on both studied parameters (NB1 duration and gap duration) we searched for a single combined parameter that could characterize the observed transitions independently from gap duration (GAPd) or NB1 duration (NB1d) alone. The ratio between GAPd and NB1d (R_G-N_ = GAPd/NB1d, see Table 1) was such a parameter along which the ABR classes were grouped together with a steep nonlinear transition between them for R_G-N_ ~ 0.5–0.6 (Fig. 2a). Differential TFRs were similar within groups, but differed greatly between groups (Fig. 2b). The observation of small within group differences, large between group differences and a nonlinear transition between the two groups along the single parameter (R_G-N_) indicates a classification of ABRs depending on the combination of the NB1 and gap durations.

**Table 1:** Overview of the grouping parameter of the ratio of gap duration to NB1 duration

		NB1 durations			
		5	10	30	100
Gap durations	2	0.4	0.2	0.07	0.02
	3	0.6	0.3	0.1	0.03
	5	1	0.5	0.17	0.05
	10	2	1	0.33	0.1

There were two conditions with the same gap/NB1 duration ratio (gap duration—5 ms and NB1 duration—5 ms; gap duration—10 ms; and NB1 duration—10 ms). Even having different gap and NB1 durations, they yielded very similar responses. LFB as well as HFB contributions in differential TFRs for these conditions were similar and if pooled together the variability of these pooled groups did not differ from that of all remaining groups. This provides per se a strong indication that the gap/NB1 duration ratio is the proper grouping variable and not the duration of gap or NB1 alone.

### Clustering analyses confirm the existence of two groups

To confirm the existence of the observed categories in the ABRs, a support vector machine supervised classifier with 10-fold cross validation was used. As input parameters for the classifier, the powers of all three frequency bands in the time window expressing the main power change in TFRs (post-stimulus time interval of 3–5 ms) were used. The trained model could classify the groups with an accuracy of 96.0% (94.4% after cross-validation) and a precision of 93.3% (86.7% after cross-validation) for the balanced/enhanced group (Fig. 2c).

**Fig. 3:**
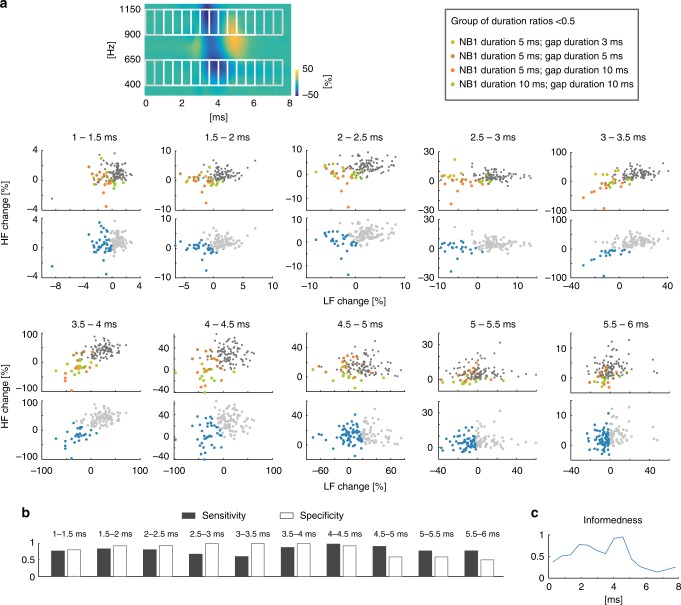
Classification (k-means clustering) of the ABR signals to the temporal sequence of the leading NB1 and trailing NB2 based on frequency power change. **a** Automatic classification of ABRs in the relation between mean low (LF) and high frequency (HF) power changes in differential TFRs for 0.5 ms bins using a k-means clustering algorithm. The rectangles in the example of a differential TFR show areas for computation of mean ‘LF change’ and ‘HF change’ variables. Scatter plots are shown for bins between 1 and 6 ms post-stimulus time (white rectangles). Upper panels of scatter plots show ‘HF change’ to ‘LF change’ relation for the group of GAPd/NB1d ratios larger than 0.5 in color (color code, see inlay) and the remainder in dark gray. Lower scatter plots show the results of the k-means clustering procedure, which automatically separates all populations into two groups. **b** Evaluation of the results of k-means clustering. Sensitivity (true positive rate) and specificity (true negative rate) are shown for bins between 1 and 6 ms post-stimulus time. **c** The time dependency of informedness (=sensitivity + specificity-1). It evaluates a level of confidence of automated clustering results. Informedness can be interpreted as a probability of informed prediction of the clustering (see Methods for details)

In order to identify which structures contribute the most to the described classification of the ABRs, the changes in the HFB and the LFB of the differential TFRs were further analyzed in 0.5 ms bins. K-means clustering, as an unsupervised clustering algorithm, was applied to the dataset in order to partition the whole population into two separate clusters with data points having the smallest distance to a common mean of each cluster. A silhouette analysis of the data confirmed two to be the optimal number of clusters (Fig. 2c). The sensitivity, specificity, and their combination—informedness (a parameter indicating a probability of informed prediction of the clustering, see Methods) were computed for each time bin and used to evaluate an accuracy of the unsupervised K-means clustering for the identification of the balanced/enhanced and the suppressed group (Fig. 3). This classification revealed that a good separation of features for the two groups (specificity > 80%; informedness > 0.5) was found for latencies between 1.5–4.5 ms. This time window corresponds to ABR waves II–IV, which have their activation sources in the cochlear nucleus and superior olivary complex, respectively^22^. This corresponds also to the observed changes of these peaks in the ABR waveforms when analyzing the ABRs in the time-domain (see Supplementary Fig. 1).

## Discussion

The results presented here provide evidence that a sequence of two sounds separated by a brief gap evokes a combination of onset ABRs that can be grouped along the ratio of the gap duration to the initial sound duration. The transition between the two groups is steep and nonlinear indicating a categorization of these subcortical responses. Consequently, we propose that the initial categorization of rapid temporal sequences occurs already at the level of the auditory brainstem. Cortical networks subsequently refine and contextualize the stimulus classification existing at subcortical levels, leaving more time and neuronal resources for complex integration of uni- and multimodal object representations.

We have shown that a release from suppression and/or enhancement of the trailing response depends on the duration of the leading noise burst (Fig. 1). Leading noise bursts of shorter duration caused an earlier transition between the response classes than leading sounds of longer duration (for NB1 = 5 ms, the transition occurred at gap durations between 2–3 ms; for NB1 = 10 ms, the transition occurred at gap durations between 5–10 ms). This observation is qualitatively similar to differences in the perceptual boundary of the transition between voiced and unvoiced phonemes of different consonants (‘p-b’ pair ~20–25 ms, ‘t-d’ pair ~30–35 ms, and ‘k-g’ pair ~40–45 ms^11^). Based on our results, one would predict that consonants with longer duration require longer voice-onset times for perceptual change from the unvoiced to the voiced phoneme. This, indeed, corresponds to the temporal features of these phonemes^23^.

**Fig. 4:**
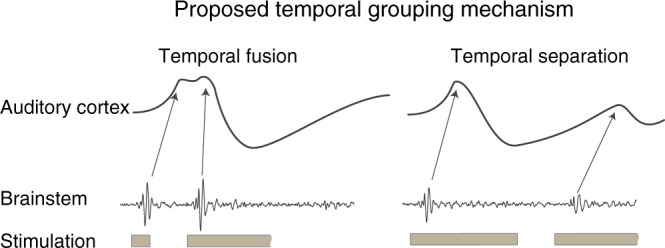
Proposed mechanism for categorical processing based on temporal contrast enhancement in the auditory system. Proposed mechanism for classification enhancement of temporal sequences in the auditory cortex. A sequence of two strong onset responses in the brainstem could temporally fuse and merge into a single (perhaps double peaked) response in the cortex^15^. On the other hand, a smaller trailing response in the brainstem can increase a temporal gap between leading and trailing response onset along auditory pathways resulting in two clearly discernable separate responses at the level of the auditory cortex^15^. Similar mechanism could be applicable also in other modalities with different time constants

The data presented here could also explain the single and double onset responses reported in the auditory cortex for voiced and unvoiced phonemes, respectively^24^. Single onset responses in the cortex could result from the temporal fusion of two strong onset responses in a fast sequence (as in the balanced/enhanced group). An amplification of the ABR_2_ would also decrease the latency of this response and, thus, facilitate a fusion of both onset responses in the cortex (Fig. 4, compare with Fig. 3 in ref. ^15^). A smaller second response in the double onset responses in the primary auditory cortex (see e.g. Fig. 3 in ref. ^15^) indicates preservation (or even amplification) of the second (trailing) response if it is temporally well separated but suppressed at the level of the brainstem (as in our suppressed group). We hypothesize that mechanisms of temporal fusion and temporal separation of the activation sequence along subcortical pathways could facilitate neuronal temporal contrast at the level of the cortex, and thus enhance perceptual categorization as well^25^ (Fig. 4).

The finding that categorical processing starts already early in the auditory pathways indicates that an important part of it occurs already at a pre-attentive level. This preparing phase could play a crucial role for the final categorical processing in the cortex, and therefore can importantly influence categorical perception. Many studies have shown that speech perception, which is based on categorical processing, deteriorates with peripheral hearing loss and/or compromised temporal processing at the periphery of the auditory system^26–28^. These observations strongly indicate an existence of a pre-attentive phase of categorical processing. We identify here the neurophysiological correlate of such pre-attentive categorical processing in the auditory brainstem that is based on temporal information.

Previous studies suggested a link between enhanced subcortical auditory processing and improved speech perception^19,29–31^. However, this view remains controversial, e.g., the categorical perception of vowels in humans is not accompanied by categorical classification at the level of the brainstem^32^. This discrepancy could be caused by different strategies that the auditory system uses for the neural coding of different speech elements. Vowels are mostly coded via spike counts, whereas consonants seem to be coded by spike timing^33^. Our study was focused on the effect of the temporal sequence of auditory stimuli in brainstem auditory processing, as it can be found in consonant vowel combinations. We have shown that the timing and duration of the stimuli in a short sequence play a crucial role in the classification of the temporal stimulus parameters (Fig. 2). This observation is consistent with the notion of importance of spike timing in speech encoding where consonants and short gaps play a crucial role^33^.

Identification and classification of ABRs into two groups based on the temporal sequence of the stimulation is an important part of the study. Therefore, we critically analyzed data using several clustering approaches (See Methods and Results for details) in order to confirm that the identified groups were not arbitrarily chosen. The classification into two clusters was optimal as proved by Silhouette analysis, which is a tool designed to test for optimal number of groups that represent the data (Fig. 2c, right panel). An automated clustering using supervised support vector machine classifier with 10-fold cross-validation confirmed very reliable classification into the two groups and their separation was close to perfect (Fig. 2c). The results of the unsupervised k-means clustering have shown a substantial degree of overlap between a label free k-means clustering and our classification along the gap/NB1 duration ratio axis. An unbalanced representation of the data between the groups (12 vs 4 conditions) could potentially bias our results. However, the high similarity within the groups, the large, highly significant difference between groups (*p* = 1.1 × 10–13 for LF band, *p* = 4.5 ×10–14 for LF band) and the high reliability of several clustering approaches strongly indicate that such a bias would not substantially influence our outcomes. Based on these results we are confident that the classification was not arbitrary, but captures the key characteristics of our data.

Other approaches to study temporal processing are paired click paradigms (e.g., refs. ^34–36^) or forward masking paradigms^37^. Our approach is different from a paired click paradigm where click stimuli follow in a much faster sequence (0.15–10 ms) when compared to our study (7–110 ms). Such a fast stimulus sequence leads to the direct interaction of the onset ABRs in the majority of the studied conditions. In our study, the minimal time interval between the onset responses was 7 ms (Supplementary Figure 2) ensuring that a relevant portion of the onset response of the first stimulus was already terminated. Consequently, the direct interaction of the onset responses plays a limited role in our experiments. Furthermore, in contrast to clicks, the duration of the noise bursts allows not only for an onset response, but also for an ongoing and offset response. The ongoing and offset responses to NB1 might influence the following onset response to NB2. Indeed, a dependence of the recovery of the trailing response on the duration of the first stimulation was observed in the forward masking paradigm^37^. Additionally, the importance of the offset responses was recently shown for temporal processing in the mouse auditory thalamus^38^. We hypothesize that depletion and availability of Ca^2+^ and potentially other ions may be involved in the observed mechanism^39^.

Here, we have described noninvasively recorded brainstem activation patterns that can form the basis of complex categorical processing of temporal features of an acoustic stimulus. In contrast to the higher-level neuronal processing, the pre-attentive processing does not critically depend on anesthesia^40^. Anesthetics influence preferentially top-down and not bottom-up activations^41^ that were the main focus in this study. Anesthesia also has been shown to influence peripheral neuronal processing substantially but only at high concentrations^42^. In the present study, the anesthesia was kept at the lowest surgical level and monitored by continuous ECG and end-tidal CO_2_ levels. Furthermore, temporal processing seems not to be critically influenced by anesthesia up to the level of the thalamus^38^. Consequently, we do not expect that anesthesia was a critical factor in the present study.

While we did not ourselves performed the behavioral experiments, it has been repeatedly shown in various species that animals, including rodents, are behaviorally able to categorize voice-onset-time based stimuli similarly to humans^10–12^. Further studies are, however, needed to reveal the exact underlying neuronal mechanism and the behavioral relevance of the observed findings. For a complete understanding of the observed phenomena, information about single neuron activities in all important auditory nuclei and the transformation of auditory activation patterns along the auditory pathway up to the level of auditory cortex is necessary. Moreover, detailed knowledge of the neurophysiological mechanisms underlying complex processing of temporal structures in the auditory system can lead to designs of behavioral training protocols that could improve categorization of acoustic features based on their temporal structure. Improved categorization would enhance recognition and perception of sound signals (e.g., phonemes), which are based on these features. Results of such studies would be of high translational value and could improve rehabilitation strategies in patients with hearing impairments.

In conclusion, the present study provides evidence that auditory brainstem processing initiates mechanism of categorical separation based on the temporal structure of the sound. We have identified two parameters that crucially affected this categorization: (1) the duration of the initial sound, and (2) the duration of the following gap before the second sound onset. Similar sound sequences form a basis for categorization of phonemes based on the voice-onset time and therefore are connected directly to speech perception. These results, together with behavioral evidence for categorical perception of vocalizations in animals^10^, further strengthens the old evolutionary origin of basic networks for phonetic analysis, and suggests that some phonetic categorical boundaries are not arbitrarily drawn but depend on phylogenetically old circuits. From the evolutionary perspective, it is easier and more efficient to reuse and refine preexisting neuronal circuits, which are already optimized for sound communication in animals, for the development of speech and language, than to develop a new network for this task.

## Methods

### Animals

In this study, eight male Dunkin Hartley guinea pigs (Charles River, Saint-Germain-sur-l’Arbresle, France; age of 6.5 ± 1.2 weeks) with a weight between 350–665 g were used. All experiments were performed in accordance to the German “law on protecting animals used for experimental purposes” as well as the European Council directive 2010/63/EU. All experiments had been approved by the local ethics committee (Animal Welfare Service at Lower Saxony State Office for Consumer Protection and Food Safety, approval number: 14/1548) and by the institutional Animal Care and Research Advisory Committee.

### Anesthesia

All experiments were performed under general anesthesia. To prevent disturbances of the gastro-intestinal tract the animals received 0.5 g BeneBac^®^ Gel (Albrecht GmbH, Aulendorf, Germany) per os (p.o.) ca. 30 min prior to induction of anesthesia. To reduce possible anxiety, animals were administered 0.3 mL diazepam (Ratiopharm, Germany) p.o. at the same time.

The anesthesia was induced via an intramuscular (i.m.) injection of a combination of 50 mg/kg ketamine hydrochloride (Ketamin 10%, WDT, Garbsen, Germany) and 10 mg/kg xylazine hydrochloride (Xylazin 2% Bernburg®, Medistar Arzeneimittelvertrieb GmbH, Ascheberg, Germany). The anesthesia was maintained with repeated injections of 1/4^th^ to 1/3^rd^ of a mixture of 50 mg/kg ketamine hydrochloride and 5 mg/kg xylazine hydrochloride. To prevent broncho-secretion, 0.1 mg/kg atropine sulfate (B. Braun Melsungen AG, Melsungen, Germany) was added to the first injection. Fluid substitution was administered via subcutaneous (s.c.) injections of Ringer solution (~2 mL/2 h). To avoid hypothermia, the animals were placed on a heating pad coupled with an intrarectal thermo probe. Topical application of dexpanthenol cream (Bepanthen^®^, Bayer Vital GmbH, Leverkusen, Germany) was used to avoid dry eyes. The animals were ventilated with room air via an intratracheal tube. For analgesia the animals received an s.c. injection of 2.5 mg carprofen (Pfizer GmbH, Berlin, Germany). Continuous monitoring of heart rate, body temperature, respiratory pressure, and end-tidal CO2 concentrations as well as toe-pinch-reflex were used to assess anesthesia depth.

### Stimulation and ABR recordings

Stimulation and recording of the auditory brainstem response (ABR) signals were performed using the AudiologyLab system (Otoconsult, Frankfurt a. M., Germany) in a sound-proof recording booth. The stimuli were presented by a calibrated loudspeaker (DT48, BeyerDynamic, Heilbronn, Germany) via a plastic cone placed in the outer ear canal. The ABR signals were recorded using subcutaneous Ag/AgCl electrodes, which were placed as follows: recording electrode—caudo-ventral of the respective pinna, reference—vertex, ground—neck. The signals were amplified (100,000×), band-pass filtered (200–5000 Hz), and recorded at a sampling rate of 100 kHz.

First, the hearing threshold of both ears was determined using clicks (duration: 50 µs, recording interval: 33 ms, 120–40 dB Attenuation, 5 dB steps). Further recordings were performed on the side with lower hearing threshold or the side last stimulated, if hearing threshold was equal on both sides (right: *n* =2; left: *n* = 6).

The sequence of noise burst stimuli (Fig. 1a) contained two noise bursts (random noise) with 1 ms rise/fall time separated by a silent gap of varying durations (2, 3, 5, and 10 ms). The leading noise burst (NB1) duration was set to be 5, 10, 30, or 100 ms; the trailing noise burst (NB2) duration was fixed at 50 ms. All noise burst sequences were presented 600 times (300 times with inverse polarity) at 30 dB above click ABR threshold. The stimulation was presented at time intervals >150 ms (151–160 ms).

### ABR analysis

The recorded data were analyzed using custom codes in Matlab (The MathWorks, Inc., Natick, USA). For all analyses, the ABR signals were off-line filtered (300–3000 Hz) using zero-shift filtering (‘filtfilt’ function in Matlab). Only the ABR responses to the onset of the respective noise bursts were analyzed in a 8 ms post-stimulus time window.

### Time-domain parameters

The quantitative parameters were collected from a time window from 1–7 ms post-stimulus onset comprising all dominant onset ABR waves. RMS values of the respective responses was computed and their ratio was calculated in order to determine the overall power relation between the onset ABR response to NB1 and to NB2. RMS ratios higher than 1 indicate stronger brainstem activation evoked by NB2 than by NB1. A similar ratio of the most dominant peak III (peak to trough) was also calculated.

### Time-frequency representations

Time-frequency representations (TFRs) of the onset ABR responses were computed to assess the synchronization of the activation of the brainstem structures. Higher frequencies indicate faster and highly synchronized activation of auditory brainstem structures, lower frequencies represent slow potential shifts indicating conditioning of the neuronal circuits by small depolarization or hyperpolarization at the time of the second activation. The Wigner–Ville distribution (2048-point precision) was used for computing the TFR for its excellent resolution both in time and frequency domains without a need of trade-off between time and frequency resolution. Cross-terms in Wigner–Ville representations were suppressed using the Choi–Williams filtering procedure^43^. Differential time-frequency representations (acquired by simple subtraction of individual TFRs) were computed to visualize the differences between the initial and trailing onset ABR response.

In order to quantitatively evaluate relative contributions of the different frequency bands, the TFR was divided into three frequency bands:

1. Middle frequency band (650–900 Hz). The ABR waves have a typical duration between 1.1 ms and 1.5 ms corresponding approximately to frequencies between 900 Hz and 650 Hz, respectively^44^. This frequency range therefore builds up the main ABR waveform;
2. Low frequency band (400–650 Hz, corresponding to wave periods of ~2.5–1.5 ms, respectively). The power of signals in this frequency band represents slower processes of neuronal activation, mainly postsynaptic potentials^45^. It can reflect modulatory processes that affects synchronization of activation sequence;
3. High frequency band (900–1150 Hz, corresponding to a wave periods of ~1.1–0.8 ms, respectively). An increase in power in this frequency band indicates higher temporal synchronization of action potentials in the brainstem^45^.

As the dominant power of the ABR signal lied within the range of 650–900 Hz with the frequency band width of 250 Hz, the low and high frequency bands were set to keep the same frequency width below and above the middle frequency range.

Temporal dynamics of the ABR signal power in the different frequency bands were evaluated by binning the differential TFRs into 0.5 ms long time segments (Fig. 3a). Then, mean differential TFR power was computed for each time-frequency bin yielding a time-frequency change representation of the individual segments.

### Clustering analysis

Support vector machine (SVM) clustering: Time-frequency changes in high and low frequency bands in post-stimulus time interval of 3–5 ms were used as input data for a supervised binary (two group) SVM classifier. The data were labeled for the Suppressed group (R_G-N_ ≤ 0.5) and Balanced/enhanced group (R_G-N_ > 0.5), respectively. SVM classifier model was then trained and 10-fold cross-validated in Matlab.

K-means clustering: Time-frequency change segments in high and low frequency bands were used as a dataset for k-means clustering (Fig. 3). The optimal number of clusters for k-means clustering was specified by evaluating the clustering solution for 2–10 clusters based on Silhouette values (a measure of data similarity to its own cluster compared to the other clusters). This analysis identified two clusters as an optimal clustering solution for k-means clustering (Fig. 2c, right panel). In the present study, a cosine distance measure as it is implemented in the Matlab ‘kmean’ function was used.

K-means clustering for two clusters was performed to validate the classification based on the R_G-N_ = NB1d/GAPd (Suppressed group: R_G-N_ ≤ 0.5; Balanced/enhanced group: R_G-N_ > 0.5). A larger cluster was considered to represent the Suppressed group as more stimulus configurations have R_G-N_ ≤ 0.5. Similarly, a smaller group was considered to represent Balanced/enhanced group. A confusion matrix was computed yielding true-positives (TP; points from putative Balanced/enhanced group clustered in the smaller cluster), false-negatives (FN; points from putative Balanced/enhanced group clustered in the lager cluster), true-negatives (TN; points from putative suppressed group clustered in the lager cluster), and false-positives (FP; points from putative suppressed group clustered in the smaller cluster). Based on the confusion matrix, the sensitivity (SE = TP/(TP + FN), called also true-positive rate), the specificity (SP = TP/(TN + FP)) and its combination informedness (I = SE + SP-1) was calculated. Informedness can be interpreted as a probability of informed prediction of the clustering result^46^, i.e., the quality of the clustering. The informedness = 1 implies that the k-means clustering resulted in the correct separation of the observed groups.

### Statistics and reproducibility

For statistical comparison of TFRs, the paired sample permutation test based on *t*-statistics (two-sided) corrected for multiple comparisons was used^47^ (*p* < 0.05). The ABR waveforms were compared using Wilcoxon rank sum test (two-sided) with false discovery rate correction^48^ (*p* < 0.01). Two-way ANOVA was performed to test statistical differences in time-domain parameters with post-hoc multiple comparison correction (*p* < 0.05).

### Reporting summary

Further information on research design is available in the Nature Research Reporting Summary linked to this article.

## Supplementary information

Supplementary Information

Description of Additional Supplementary Files

Supplementary Data 1

Reporting Summary

## Data Availability

The data that support the findings of this study are available from the corresponding author upon reasonable request. All averaged individual recorded ABR traces for all recorded conditions are shown in Supplementary Fig. 2. The source data underlying Fig. 2 are shown in Supplementary Data 1.

## Code availability

The custom computer codes used in this study are available upon reasonable request to the corresponding author.
