# Supplementary material for: Categorical processing of fast temporal sequences in the guinea pig auditory brainstem
Source: Commun Biol. 2019 Jul 19;2:265. doi: 10.1038/s42003-019-0472-9 (PMC6642126; doi:10.1038/s42003-019-0472-9)
Supplement: Supplementary file 1 — Supplementary Information [file 42003_2019_472_MOESM1_ESM.docx]

SUPPLEMENTARY INFORMATION

**
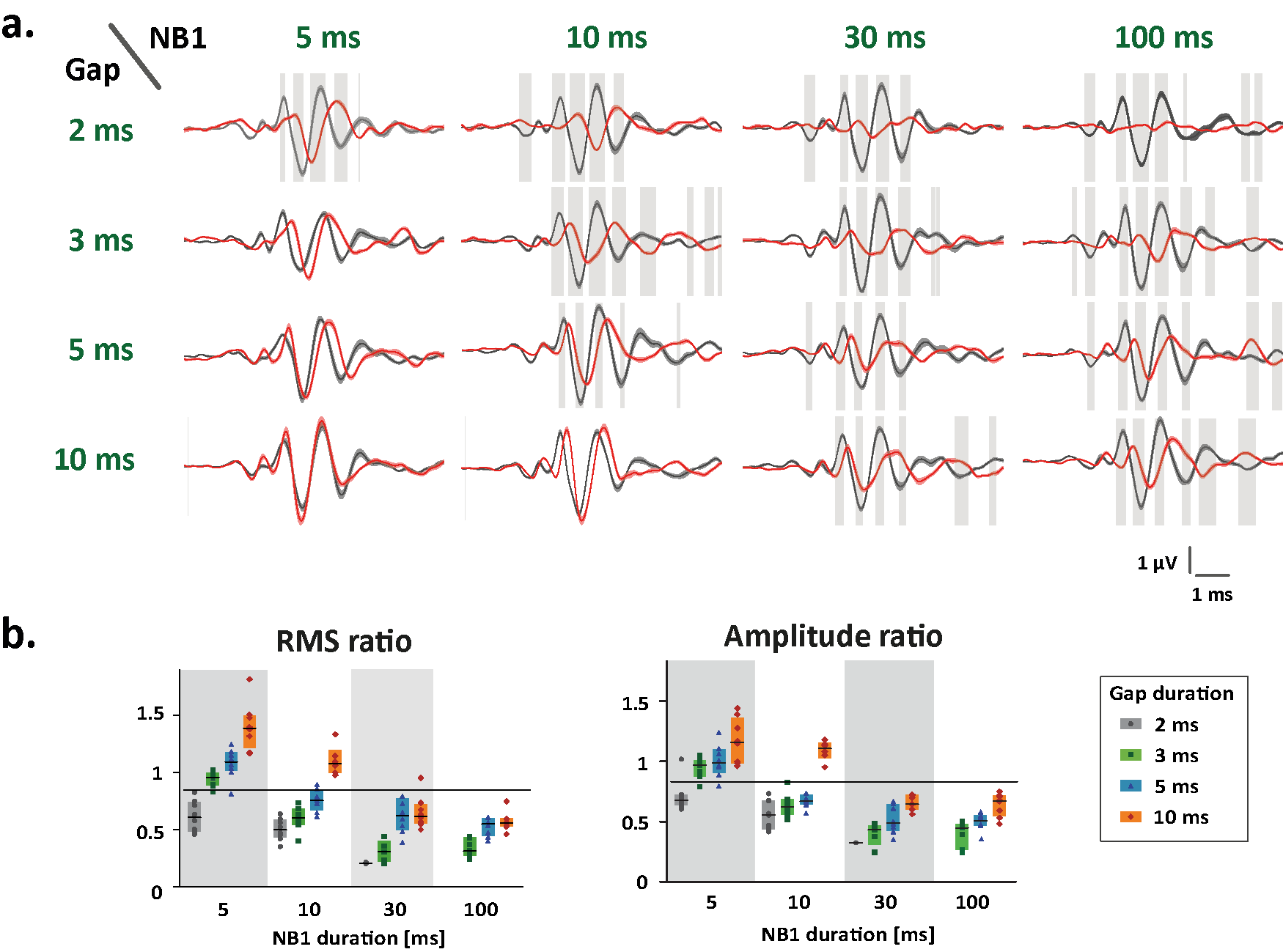
**

**Supplementary Figure 1. Time representations of the onset ABRs to the leading and trailing noise burst and their parameters**

1. Grand means and standard errors of all onset ABRs evoked by the leading NB1 (gray traces) and the trailing NB2 (red traces) for all recorded combinations of NB1 and gap duration. The responses are overlaid relative to the onset of the noise bursts. For all responses, a poststimulus time window of 8 ms is shown. The background shading indicates statistically significant different activations (p<0.01; multiple comparison corrected).
2. The ratio of ABR_2_/ABR_1_ for both root-mean-square (left) and peak amplitude of ABR wave III (right) shows a suppression for ratios of the gap and NB1 duration ≤ 0.5 and an either balanced or enhanced response for R_G-N_ > 0.5. The horizontal lines separate the two groups accordingly to the cluster analysis. A 6 ms analysis window was used. Boxes indicate 25/75% quartile.

#
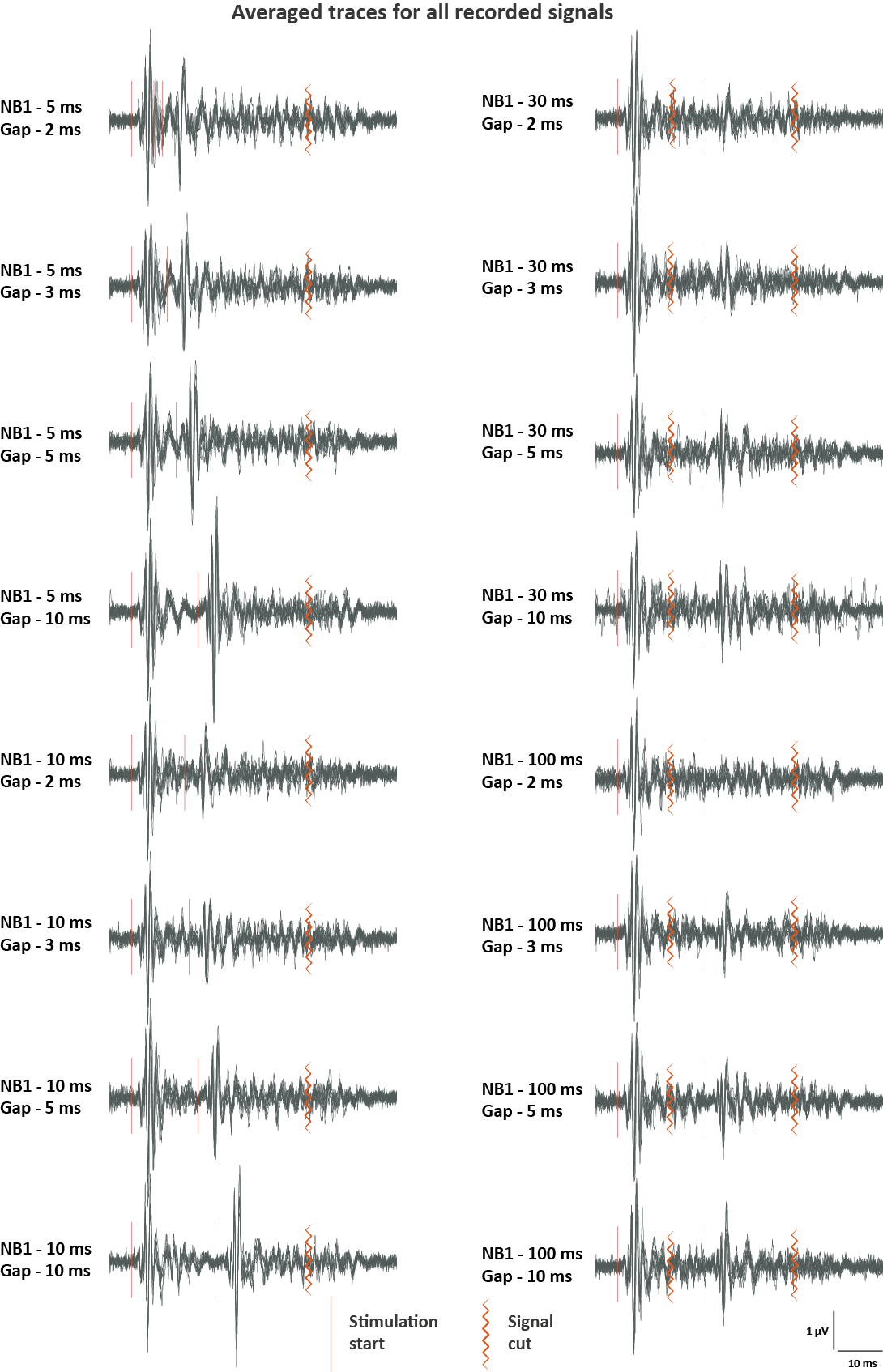


**Supplementary Figure 2. Averaged traces of the raw recorded signals for all individual animals**

All averaged individual recorded ABR traces for all recorded conditions. Vertical red straight lines indicate stimulation (noise burst) begin. In order to visualize the long signals on a limited space, the middle parts of the ongoing responses evoked by long stimulations (durations > 10 ms) were cut out. Red vertical zigzag lines indicate the time points where the signals were cut.
