# Supplementary material for: Categorical processing of fast temporal sequences in the guinea pig auditory brainstem
Source: Commun Biol. 2019 Jul 19;2:265. doi: 10.1038/s42003-019-0472-9 (PMC6642126; doi:10.1038/s42003-019-0472-9)
Supplement: Supplementary file 2 — Description of Additional Supplementary Files [file 42003_2019_472_MOESM2_ESM.pdf]

Description of Additional Supplementary Files

File Name: Supplementary Data 1

Description: Relative power of TFR differences between ABR_1 and ABR_2 in low and high frequency bands (400-650 Hz and 900-1150 Hz, respectively) for latencies of 3-5 ms (Excel file).
